# Adaptation and Validation of the Hydration Status Questionnaire in a Spanish Adolescent-Young Population: A Cross Sectional Study

**DOI:** 10.3390/nu11030565

**Published:** 2019-03-06

**Authors:** Ana Isabel Laja García, Maria de Lourdes Samaniego-Vaesken, Teresa Partearroyo, Gregorio Varela-Moreiras

**Affiliations:** Department of Pharmaceutical and Health Sciences, Universidad CEU San Pablo, 28668 Madrid, Spain; a.laja90@gmail.com (A.I.L.G.); l.samaniego@ceu.es (M.d.L.S.-V.); t.partearroyo@ceu.es (T.P.)

**Keywords:** hydration status, water balance, questionnaire, validation, adolescents-young

## Abstract

The achievement of adequate hydration status is essential for mental and physical performance and for health in general, especially in children and adolescents. Nevertheless, little is known about hydration status of this population, mainly due to the limited availability of research tools; thus, the objective of the current study was to adapt and validate our hydration status questionnaire in a Spanish adolescent-young population. The questionnaire was validated against important hydration markers: urine colour, urine specific gravity, haemoglobin, haematocrit and total body water and involved 128 subjects aged between 12–17 years. Water intake was also estimated through a three-day dietary record and physical activity was assessed through accelerometers. Participants completed the questionnaire twice. Water balance and water intake were correlated with urine specific gravity and with total body water content. Water intake obtained by the questionnaire was correlated with results from the three-day dietary record. The intraclass correlation coefficient indicated moderate concordance between both recordings and the Cronbach’s alpha revealed high consistency. The Bland and Altman method indicated that the limits of agreement were acceptable to reveal the reliability of the estimated measures. In conclusion, this is the first time that a questionnaire is valid and reliable to estimate hydration status of adolescent-young populations.

## 1. Introduction

Water is the main component of human body and is essential for life and health. It is crucial for the adequate function of several physiological processes, in such a way that an inadequate hydration status (HS) is associated with poor health [1,2].

The state in which total body water is insufficient for proper cell, organ and system functioning is called dehydration [3]. It is well known that grave dehydration status is associated with health problems such as confusion, delirium, impaired immune, renal and gastrointestinal functioning. But even mild dehydration states entail negative health consequences: headache, irritability, decrement in physical performance and reduced cognitive function, among others [2,4,5,6,7,8,9]. These facts gain special importance in children and adolescents considering that it could have implications for both health, sports and school performance [6,7,8,9,10,11,12]. Nevertheless, paradoxically, little is known about the HS of this population and its consequences [6].

On the other hand, in recent years, beverage intake in children and adolescents has been more widely investigated [12,13,14] and in general, available data suggest that it is suboptimal compared to reference values [15]. In this context, it is important to take into account that current guidelines for adequate water intake (WI) vary widely: many regional and global authorities have established their own recommendations; however, these recommendations are based on population median WI, with limited consideration of links between WI and HS and without links between WI and health [16,17]. The European Food Safety Authority (EFSA) has published the most recent official guideline for total WI in 2010 [16,18] and results from nutritional surveys show that its noncompliance in children ranged from 30% in Brazil, Spain and the United Kingdom, to more than 80% in France, Belgium and China [15]. These results are of concern, not only for its possible negative health impact [6,10,11,12,19] but also for it long-term behavioural effect, considering that habits are established early in life and can predict dietary intake patterns that persist during childhood, adolescence and into adult age [20]. Despite all this, no conclusion on HS can be drawn from these surveys, only that this population may be at risk of dehydration [21]. This lack of information about HS is mainly due to the complexity that its measurement implies [21,22,23,24].

The body’s fluid level is determined by water balance (WB), defined as the equilibrium between water input and output [3]. It is a dynamic process, which is influenced by several variables (environmental conditions, food and drink availability, physical activity, thirst and hormonal mechanisms between others) and is precisely regulated over a 24 h period, as intake and losses must be equal in such a way that under condition of temperate ambient temperature and with a moderate activity level it remains relatively constant [1,3]. The accurate measurement of HS is complicated because body water turnover occurs constantly and water moves between intracellular and extracellular compartments [23]. Several biomarkers have been proposed such as blood (haematocrit and haemoglobin) and urinary indices (specific gravity, urine colour and urine osmolality) as well as the estimation of body water via bioelectrical impedance (BIA) or spectroscopy. Nevertheless, there are no markers, which have been recognized as “gold standard,” because no single method appears to be ideal for all situations [17,22,23,24,25]. Nowadays, the combination of different hydration indices seems to be the more appropriate available method for the estimation of HS.

Recently, a novel research tool, a questionnaire entitled: The Hydration Status Questionnaire (HSQ) has been developed by our research group and validated through different biomarkers in adult population [26]. The HSQ supplies information about WI, water elimination (WE) and WB and it could be applied as a screening method to detect individuals at risk of dehydration and to support adequate recommendations. The adaptation and validation of the HSQ to other population groups could allow for a better, deeper knowledge of HS.

For all the aforementioned, the aim of the present study was to adapt and validate the HSQ in a healthy adolescent-young Spanish population (HSQ-AY).

## 2. Materials and Methods

The HSQ was modified to HSQ-AY (see Supplementary File S1) to make it suitable and understandable for adolescent young population. The main modifications respect to the HSQ were a simpler and more understandable language for the target population, including images of the drinks and foods included as well as different sizes of rations to facilitate their completion and, likewise, beverages and foods were eliminated with very low frequency of consumption. After its modification, the HSQ-AY was validated in the target group as described below, with a similar methodology that was followed for the validation of the HSQ [26].

Ethical approval was granted by the Clinical Research Ethics Committee of the CEU San Pablo University (Madrid). The corresponding ethical code was 120/16/06. The study has been performed in accordance with the ethical standards laid down in the 1964 Declaration of Helsinki and its later amendments. Participants and their parents or guardians were informed on the objectives of the study and the procedures involved and both signed an informed consent prior to their inclusion in the study. All personal data are confidential and only investigators assigned to the project have access to them. In any case, it complies with the General Data Protection Regulation (2016/679).

### 2.1. Design of the Hydration Status Questionnaire in a Healthy Adolescent-Young Spanish Population

The HSQ-AY contains five sections in which the main factors that affect hydration were recorded [3,18,22,25,26,27,28,29]: (a) personal information, (b) medical history, (c) hydration habits and knowledge, (d) water beverage and food frequency questionnaire (WBFFQ) and (e) WE. WI was recorded through the WBFFQ, which included all beverages and foods with water content higher than 80%, present in the Spanish Food Composition Tables [30]. The establishment of this cut-off value in the food and beverages included is necessary to make the length of the questionnaire appropriate to complete in a period of 20-30 min and recall the main contributors to water consumption. Food and beverage items were classified into twelve groups: (d.1) water, (d.2) juices, (d.3), sodas, (d.4) milk and dairy products, (d.5) coffees, (d.6) tea and infusions, (d.7) alcoholic beverages, (d.8) other beverages (plant-based beverages and horchata), (d.9) fruits, (d.10) vegetables and (d.11) cooked dishes. Pictures of each food and beverage presented in the questionnaire as well as different household units such as glasses, bottles, cups and plates of various sizes were included in the questionnaire to facilitate its completion. The frequency of consumption was evaluated using three categories: (a) daily; (b) per week; (c) or per month and times of consumption were recorded as “at breakfast,” “at lunch,” “at dinner,” and/or “between hours.” In addition, the seasonality of fruits, vegetables, beverages and cook dishes was considered in the data processing since not all these foods are usually intake throughout the year. To estimate WE from urination and defecation, the questionnaire provided different frequency options in each case [31] (urination options: once/day, two-four times/day, five-seven times/day, eight-ten times/day and more than ten times/day; defecation options: once/day, five-six time/week, three-four times/week, one-two times/week or less than one time/each 10 days). To calculate WE from sweating, a 10-point scale was used for both, physical activity and sedentary conditions [31]. WB was calculated through WI and WE data. The other sections of the HSQ-AY allow for the assessment of the participant profile.

### 2.2. Questionnaire Analysis

The data processing was performed as described below, using the same methodology as for the HSQ [26].

The water content from beverages and foods was calculated using the Spanish Food Composition Tables by Moreiras et al. [30] and the Spanish Food Composition Data Base (BEDCA) [32]. The amount of water from drinking water, beverages and foods were calculated separately and expressed as millilitres of WI per day. Water provided by each beverage was calculated according to the following formula: millilitres of beverage consumed per day × duration of its seasonality in days/336 × water content/100. Water from foods was calculated in the same way but also considering the edible portion. To calculate WE from sweating in sedentary conditions, the 10-point scale described before was used. The duration in hours per day of this condition was multiplied with a factor to quantify WE; this factor depends on the score that the participant gave for sweating in the scale (i.e., point 1 corresponded to 0.01 mL water/h and point 10 to 0.02 mL/h), in-between values varied in a proportional manner. The WE from sweating during exercise was also estimated using the 10-point scale [31]. To calculate it, the duration and intensity level of the physical activity performed during seven consecutive days were estimated by accelerometers. The duration of physical activity in hours was multiplied with a factor that depends of the score given in the scale and of the activity intensity level: for intense exercise, point 1 corresponded to 1000 mL water/h and point 10 to 2000 mL/h, for moderate exercise point 1 corresponded to 400 mL/h and point 10 to 700 mL/h and for mild exercise, point 1 corresponded to 200 mL/h and point 10 to 400 mL/h [33,34,35,36]. In-between values varied in a proportional manner. To estimate WE from urination and defecation, participants had five frequency options in both cases. These options were transformed into a 5 point-scale, in which the first option (once/day) corresponded to point 1 and the last one (more than 10 times/day or 1 time/10 days) corresponded to point 5. For urination, point 1 corresponded to 750 mL water/day and point 5 to 2500 mL water/day. For defecation, point 1 corresponded to 150 mL water/day and point 5 to 75 mL water/day. In-between values varied in an analogous manner. WB was defined as the difference between total WI and total WE.

### 2.3. Questionnaire Validation

The validation process took place in the same range of months (April to May) of the two following years: 2017 and 2018. Volunteers were recruited from three schools in Madrid (Arcadia Private School, Secondary Institute Encinas and CEU San Pablo Montepríncipe School), Spain. The inclusion criteria were: individuals who were (a) mentally and physically healthy (b) aged 12–17 years. Exclusion criteria were those suffering from disease related to hydration, including renal impairment, urinary tract infection, WB disease, diabetes and or females who were menstruating during the study. The recruitment of volunteers was performed through informative talks gave in each school. The sample size was calculated according to Nunnally criterion [37], which recommends a ratio of minimum 5 participants for each item of the questionnaire.

The validity study was performed using several indicators of HS [22,24,26,38,39,40,41]: urine specific gravity (USG), urine colour (UC), plasma haemoglobin, haematocrit and total body water content (TBW). WI data obtained by the HSQ-AY were compared with water consumption from a three-day dietary record (3DR). In addition, haemodynamic data (pulse, systolic blood pressure (SBP) and diastolic blood pressure (DBP) were collected because these parameters are also related with HS [42]. For the reliability study, participants completed the questionnaire twice over the course of 28 days. The tests and measurements included in the validation process were performed in a facilitated room of each school by qualified staff.

### 2.4. Validation Protocol

**Figure 1:**
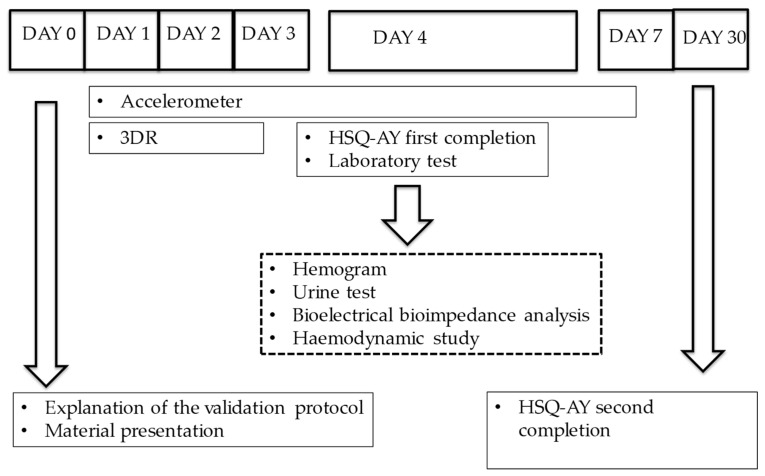
Protocol of the validation process. 3DR: three-day dietary record; HSQ-AY: hydration status questionnaire in a healthy adolescent-young Spanish population.

Each volunteer’s first visit was preceded by a short explanation on the procedures involved in the validation process and its protocol (Figure 1).

For seven days, participants wore an accelerometer that estimated their physical activity and completed, over the course of three consecutive days, (one weekend and two weekdays) a 3DR of foods and beverages. They were asked to give detailed descriptions of each food and beverage item consumed, including portion size, cooking method applied, recipe, brand and moment of consumption, providing them previously with clear instructions on how to fill in it. Subjects were also instructed to follow their usual diet. The DIAL™ software was used to process the information of the 3DR. At last, participants completed the HSQ-AY and the following laboratory tests and measurements were performed under fasting conditions:

- Haematological variables: haemoglobin, haematocrit and erythrocyte were determined by capillary finger-stick whole blood with Calligari™ Analyser.
- Body composition: TBW and water percentage was estimated by BIA with Bioscan Spectrum™ Multifrequency. Individuals were weighed using a digital scale with an accuracy of 200 g (SECA™ 877). Height was measured to the nearest 0.1cm using a wall-mounted stadiometer (SECA™ 213). The anthropometric measurements were made according to the recommendations of the International Standards for Anthropometric Assessment (ISAK) [43] by level I and II accredited anthropometrists.
- Urine variables: volunteers provided a first morning urine sample in which urine pH and USG was determined using urine stick test Spinreact™ and urine colour via the Urine Colour Chart [44]. Results were compared with reference values of hydration biomarkers in first urine morning spot established by Armstrong et al. [40] (Euhydration: specific gravity = 1.023–1.025, urine colour = 4–5).
- Haemodynamic variables: pulse, SBP and DBP were determined using a digital sphygmomanometer (Omron™, M3 model).

### 2.5. Statistical Analysis

Results are presented as mean and 95% confidence interval. Differences between variables were assessed with the Student’s paired *t*-test and considered significant at *p* < 0.05. The validity of the questionnaire was evaluated using Pearson’s (*r*) coefficient to estimate the correlation between WI, WE and WB with quantitative discrete variables (haemoglobin, haematocrit, USG, TBW, pulse, SBP, DBP) and Tau b the Kendall (τ) for ordinal qualitative ones (UC). Test-retest reliability was assessed using the intraclass correlation coefficient (CCI) to demonstrate that results were consistent over time. The Bland-Altman plot was used to represent graphically the agreement between measurements in both administration of the questionnaire. Moreover, Pearson’s coefficient between the difference and the average of the variables estimated through both records was calculated to assess potential bias in estimation (significant values of Pearson’s coefficient indicate divergence in the variable between the two administrations). Student *t* test was applied to further evaluate the difference between the two recordings. Cronbach’s alpha (α) was also applied to assess the internal consistency of the HSQ in both administrations. All statistical analyses were performed using SPSS 24.0 Software (IBM Corp., Armonk, NY, USA).

## 3. Results

### 3.1. Sample Characteristics

**Figure 2:**
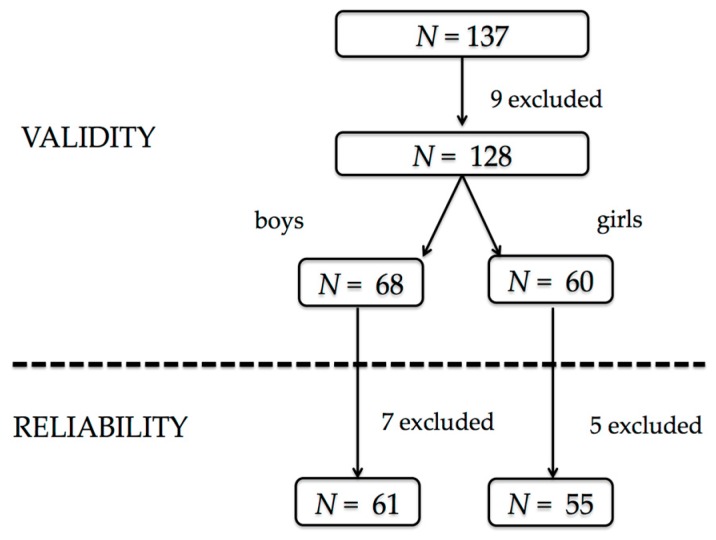
Effective sample size.

A total of 137 volunteers were recruited for the validation process: nine of them were excluded after meeting some of the exclusion criteria and twelve participants did not complete the questionnaire a second time. Therefore, the final sample size for the validity study was 128 participants, 68 males (53.1%) with a mean of 14.1 (13.8–14.4) years and 60 females (46.9%) with a mean age of 14.4 (14.0–14.8) and 116 for the analysis of the reproducibility, 61 males (52.6%) and 55 females (47.4%) (Figure 2).

Their anthropometric characteristics, body water content, urine and blood markers and haemodynamic data are presented in Table 1.

As it can be observed in Table 1, average values for TBW were lower in females than in males. With the exception of the urine pH, there were no significant differences in urinary indices between both genders. Haematological indices (haematocrit and haemoglobin) were higher in males than in females and no differences were found in the quantity of erythrocytes. Al last, blood pressure values of males were higher than in females and no differences were found in the pulse between both genders.

Results of the HSQ-AY, sorted by gender, are presented in Table 2.

As shown, except for WE, there were no significant differences in any variable between both genders. This difference is mainly due to the elimination of water through sweat, which is higher in males than in females. Results using accelerometers information showed that in males WE through sweat was 2302.5 (2138.5–2466.5) mL and 1620.3 (1465.8–1774.7) mL (*p* = 0.000) in females, while no differences were found in WE through urination (*p* = 0.115) and defecation (*p* = 0.082). This fact may be caused by the intensity and duration of the physical activity practiced by each gender.

According to the results obtained from 3DR, total WI of the sample was 2297.1 (2170.1–2424.1) mL/day. Sorting results by gender, total WI of males was 2280.0 (2103.4–2456.5) mL/day and 2316.5 (2128.4–2504.6) mL/day in females.

### 3.2. Validity of the Questionnaire

To assess the validity of the tool, WB, WI and WE estimated by the HSQ-AY were correlated with the hydration indices analysed.

Moderate agreement between the WB and the respective biomarkers was evident for USG (*r* = −0.202, *p* = 0.023). The total WI was correlated with this same biomarker (*r* = −0.184, *p* = 0.037) and, also, with the haematocrit value (*r* = −0.231, *p* = 0.009) and with the TBW (*r* = 0.263, *p* = 0.003). Haemodynamic parameters were correlated with WE: Pulse, SBP and DBP were correlated with WE through sweat (*p* = −0.237, *r* = 0.000; *r* = 0.178, p = 0.045; *r* = −0.281, *p* = 0.001, respectively) and with total WE (*r* = −0.189, *p* = 0.032; *r* = 0.200, *p* = 0.024; *r* = −0.209, *p* = 0.018, respectively). At last, drinking water, water from beverages, total WI and WB estimated through the HSQ-AY were correlated with WI from 3DR (*r* = 0.539, *p* = 0.000; *r* = 0.504, *p* = 0.000; *r* = 0.468, *p* = 0.000; *r* = 0.357, *p* = 0.000).

Results analysed by gender showed that WB and total WI were correlated with USG (*r* = −0.315 *p* = 0.014; *r* = −0.306, *p* = 0.017) among females and with TBW in males (*r* = 0.387, *p* = 0.001; *r* = 0.392, *p* = 0.001). UC was correlated with WE through urine only among females (τ = −0.228, *p* = 0.042). In males, blood indices were correlated with total WE (Haemoglobin: *r* = −0.239, *p* = 0.050; Haematocrit: *r* = −0.282, *p* = 0.020) and with WE from sweat (Haemoglobin: *r* = −0.272, *p* = 0.025; Haematocrit: *r* = −0.316, *p* = 0.009). With respect to haemodynamic parameters, in males DBP was correlated with total WE (*r* = −0.382, *p* = 0.001) and with WE through sweat (*r* = −0.404, *p* = 0.001), while in females the correlation obtained was for SBP with total WE (*r* = 0.282, *p* = 0.029). At last, as well as for the whole sample, drinking water, water from beverages and total WI estimated through the HSQ-AY were correlated with WI from 3DR in both, males (*r* = 0.470, *p* = 0.000; *r* = 0.424, *p* = 0.000, *r* = 0.398, *p* = 0.001, respectively) and females (*r* = 0.624, *p* = 0.000; *r* = 0.609, *p* = 0.000; *r* = 0.588, *p* = 0.000, respectively) and WB only among females (*r* = 0.505, *p* = 0.000).

### 3.3. Reliability of the Questionnaire

Results from both HSQ-AY administrations are presented in Table 3.

**Figure 3:**
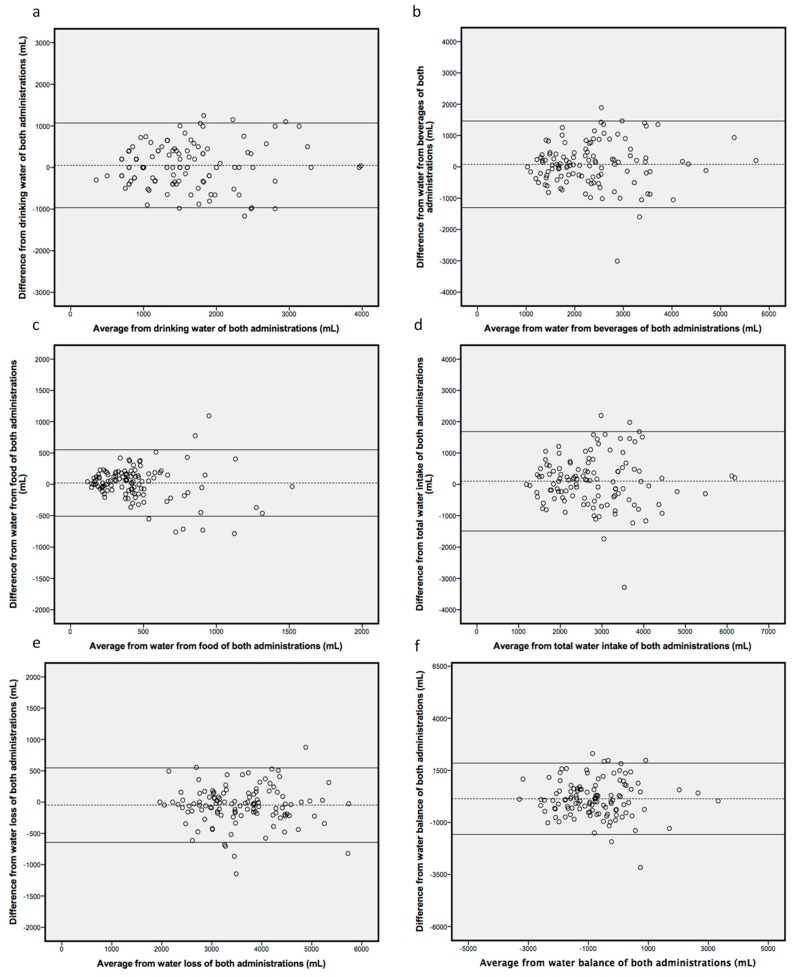
Bland-Altman plots of differences versus means for the variables: (**a**) drinking water (regression line has a slope of 0.0598 and an intercept of −44.783 (95% confidence interval: −0.06–0.18) and *p* ≥ 0.05), (**b**) water from beverages (regression line has a slope of 0.0303 and an intercept of 8.225 (95% confidence interval: −0.12–0.18) and *p* ≥ 0.05), (**c**) water from food (regression line has a slope of 0.1851 and an intercept of 104.28 (95% confidence interval: −0.38–0.01) and *p* ≥ 0.05), (**d**) total water intake (regression line has a slope of 0.189 and an intercept of 153.22 (95% confidence interval: −0.18–0.13) and *p* ≥ 0.05), (**e**) water loss (regression line has a slope of −0.0057 and an intercept of −27.662 (95% confidence interval: −0.08–0.06) and *p* ≥ 0.05) and (**f**) water balance (regression line has a slope of −0.0237 and an intercept of 117.8 (95% confidence interval: −0.17–0,12) and *p* ≥ 0.05).

To analyse the test-retest reliability, the ICC was calculated obtaining a value of 0.842. As it can be observed in Table 3 there were no differences in any variables between the two recordings. According to the Bland and Altman method (Figure 3) the mean differences of the estimated variables did not differ from zero (Student *t* test). The limits of agreement were quite narrow and all six-scatter plots were predominantly distributed within the 95% limits of agreement, as well as being considered acceptable to reveal the reliability of the estimated measures. No bias was evident regarding the two recordings in all studied cases (drinking water: *r* = 0.968, *p* = 0.335; water from beverages: *r* = 0.404, *p* = 0.687; water from food: *r* = −0.192, *p* = 0.056; WI: *r* = −0.240 *p* = 0.811; WE: *r* = −0.162, *p* = 0.871; WB: *r* = −0.318, *p* = 0.751). To test the internal consistency of the questionnaire, the Cronbach’s α of each recording was calculated, obtained as results 0.811 and 0.809 respectively.

## 4. Discussion

From our knowledge, this is the first time that a questionnaire, which allows for the estimation of WB, has been validated in a healthy adolescent-young Spanish population. Results of the validation process showed that, WB, total WI and WE were correlated with several of the most important markers of HS: USG, TBW, UC and also with haematocrit and haemoglobin. It is important to highlight that an adequate WB is not equivalent to an adequate HS if it is the result of a low WI and a low WE [45]. For this reason, it was considered that the estimation of only WB was not still enough for the assessment of HS and as consequence WI and WE should also be included in the validation of the questionnaire.

Nowadays urinary indices are the most widely hydration markers used in the field of research [17,39,40,46] and recent investigations have demonstrated that both USG are strongly correlated with urine osmolality, which has been proposed as the most promising marker of HS [23,24,39]. USG is not only useful during acute water loss but also in real life conditions, constituting an accurate and rapid indicator of HS. Nevertheless, UC is somewhat more subjective because depends on several factors such as food and/or medicines consumed and therefore it must be used in combination with a more quantifiable method [47,48,49]. In the current study WB and WI of the whole sample were correlated with USG in an inverse manner, while WE through urine of females was correlated with UC, in such a way that higher amount of water output was related to lighter colour of the urine. Another point to underline is that results obtained for USG did not differ between males and females but TWB was different between genres. This possibly is due to body composition differences between them. Specifically, males present a higher proportion of lean mass than females.

In respect to the TBW, which is an indicator of HS [22,24,39,41,46], a positive correlation was found for TWI in the whole sample and also with WB but only among males. It is known that the most accurate methodology for the estimation of TBW is the criterion isotope method such as deuterium oxide, hydrogen, tritium and oxygen-18. But these techniques are time-consuming, expensive and require cumbersome equipment and in consequence, they cannot be applied at the community level. In the current study it was estimated through BIA, which is a cost-effective, non-invasive and easy technique, which has produced valid measurements of TBW when compared to a criterion method, such as deuterium oxide [22,50,51,52,53,54,55]. Nevertheless, given its limitations, (i.e., dependence on factors such as skin temperature, body posture before the measurement) it must be performed under controlled conditions [56]. Because of this, the test was carried out with participants in fasting conditions and without practicing intense physical activity in the previous 24 h, with the recommended body posture and correct electrode positioning. Women who were menstruating were also excluded from the validation process.

On the other hand, the haematological markers (haemoglobin and haematocrit) respond to greater changes in HS, in such a way that in severely dehydration status both parameters will increase with respect to basal levels [22]. In this study, total WI as well as WE were correlated with both parameters: higher volumes of WI were related to lower values of haematocrit and higher volumes of water output with lower values of haemoglobin and haematocrit. It may be explained because in those individuals whose drink more, the WE is also greater.

At last, it is well known that HS also affects haemodynamic parameters. High blood pressure is common in people who are chronically dehydrated; nevertheless, in acute dehydration status the volume of water in the bloodstream decreases and a diminution in the blood pressure could happen [42,57]. However, haemodynamic parameters are not able to identify fluid imbalances independently of other indices. In the current study haemodynamic parameters were correlated with WE, especially with WE through sweat, which may demonstrate certain relation between the physical activity and haemodynamic parameters.

Results of the current study were in accordance with known literature showing lower median values for TBW in woman than in men [58,59]. Blood and urinary indices were within the physiological ranges [18,60] nevertheless, according to the urine colour chart [44], a score equal or higher than three indicates risk of hypohydration in children and adolescents [61]; in the present study, both males and females obtained a score higher than this value. On the other hand, blood pressure values were compared with the *Task Force for Blood Pressure in Children*; which establish for age and sex the percentiles of blood pressure values related to the height percentiles [62]. A normal blood pressure is defined as a SBP and DBP that is less than the 90th percentile for sex, age and height. According to this criteria blood pressure values of the sample included in this study were optimal.

With regard to the reproducibility of the questionnaire, the Cronbach’s α revealed a high and similar consistency in both recording (values equal or higher than 0.7 are considered adequate). The ICC revealed the existence of strong concordance between both administrations of the questionnaire (ICC higher than 0.8 indicate high concordance). The Bland-Altman method allows assessing the agreement between the methods across the range of WI and losses and can determine if there was any systematic difference between the administrations of the questionnaire and to what extent the two administrations agree (limits of agreement). Accordingly, the HSQ-AY was repeatable for all the components studied (WI, WE and WB).

At present there are few studies that evaluate WI in the target population group and most of them used unspecific questionnaires to its estimation. Recently, some studies have been developed in Spain with this objective but only one of them used a specific hydration questionnaire and it did not included water from food [15]. In general, this type of surveys tends to underestimate, as they solely record eating occasions around meals but not all drinking occasions [15,25,63,64,65]. Because of that, water consumption obtained in the current study through the HSQ-AY was slightly higher when compared to other studies; however, WI obtained through the 3DR was quite similar [15,64]. In addition, results from different epidemiological studies have shown that foods may provide the 20% [15,63,64,65,66] of total WI, being results of the present study consistent with these data for the group of females (19.2%) but not in males, whose consumption of water from food was much less, roughly a 13%.

From our knowledge, there are no hydration questionnaires specifically designed and validated in the adolescent-young population, which take into account water from all sources (liquid and solid) and WE. Nevertheless, the estimation of WE is essential to calculate WB. In the current study, the estimation of WE was performed through the use of point scales for self-estimation, which have demonstrated to be valid to achieve this objective [33,34,35,36,67]. The assessment of WE through sweat was based on the information acquired by accelerometers that participants wore for seven days. This allowed a more accurate estimation of WE, because WE through sweat depends greatly of the duration and intensity of the physical activity performed and accelerometers are at the present the most refined method to its quantification [68].

Advantages of this study included the design of the questionnaire, which has demonstrated to be understandable, attractive and suitable to estimate WI and WB in the target population. Its structure, length and colour code, as well as the inclusion of pictures of each food and beverage, has been a key aspect to achieve this objective. This fact plays a crucial role in the validity and reliability showed by the questionnaire against important indicators of HS. However, the most important limitations refer to the non-availability of 24 h urine sample as well as the measurement of USG through reagent strip.

## 5. Conclusions

The present questionnaire may be used as an affordable and valid screening tool to estimate WB in healthy children and adolescents, which would allow further a deeper knowledge of HS of the general population.

## Figures and Tables

**Table 1:** Anthropometric characteristic, body water content, urine and blood markers and haemodynamic data of participants.

	Males (*n* = 68)	Females (*n* = 60)	*p* Values
Weight (Kg)	58.3 (55.3–61.3)	54.8 (52.2–57.5)	0.084
Height (cm)	166.7 (164.5–169.0)	161.4 (160.0–162.7)	0.000
Body fat mass (Kg)	15.1 (13.6–16.6)	18.2 (16.7–19.7)	0.004
Body lean mass (Kg)	44.0 (41.9–46.1)	36.9 (35.9–38.0)	0.000
TBW (%)	56.0 (55.0–57.1)	51.3 (50.1–52.5)	0.000
TBW (L)	32.9 (31.3–34.5)	28.1 (27.1–29.1)	0.000
Specific gravity (g/L)	1.025 (1.024–1.027)	1.025 (1.024–1.026)	0.681
pH	5.3 (5.2–5.4)	5.1 (5.1–5.2)	0.043
Urine colour	3.5 (3.2–3.7)	3.4 (3.2–3.9)	0.671
Haematocrit (%)	43.1 (42.3–44.0)	40.0 (39.1–41.0)	0.000
Erythrocyte (mill/μL)	5.5 (4.1–6.9)	4.4 (4.3–4.6)	0.133
Haemoglobin (g/dL)	14.6 (14.2–15.0)	13.7 (13.2–14.1)	0.002
SBP (mmHg)	120.1 (116.6–123.5)	111.9 (107.6–116.2)	0.003
DBP (mmHg)	62.3 (60.2–64.5)	67.1 (65.0–69.2)	0.003
Pulse (beats/min)	70.9 (68.1–73.8)	81.0 (78.0–84.9)	0.000

Results are presented as mean and confidence interval. *p* values derived through the Student’s *t* test. TBW: Total body water; SBP: Systolic blood pressure; DBP: Diastolic blood pressure.

**Table 2:** Water intake from all sources, water elimination and water balance obtained by the hydration status questionnaire adolescent-young (HSQ-AY), sorted by gender.

	Males (*n* = 68)	Females (*n* = 60)	*p* Values
Drinking water (mL/day)	1897.6 (1683.2–2112. 1)	1611.1 (1293.2–1929.0)	0.138
Water from beverages (mL/day)	2713.7 (2465.0–2962.4)	2308.8 (1983.2–2634.4)	0.051
Water from food (mL/day)	424.0 (366.8–481.2)	501.5 (425.3–577.8)	0.107
Water intake (mL/day)	3137.7 (2880.7–3394.8)	2810.3 (2465.6–3154.9)	0.131
Total water loss (mL/day)	3811.0 (3631.1–3990.3)	3213.5 (3030.0–3397.0)	0.000
Water balance (mL/day)	−673.3 (−975.1–−371.5)	−403.2 (−771.7–−34.7)	0.259

Results are presented as mean and confidence interval. *p* values derived through the Student’s *t* test.

**Table 3:** Results of the reliability procedure for de HSQ-AY.

	1st Completion (*n* = 128)	2nd Completion (*n* = 116)	Mean Difference	*p* Values	Limits of Agreements
Drinking water (mL/day)	1628.1 (1472.2–1784.0)	1577.0 (1429.3–1724.7)	51.1	0.292	1068.8–966.6
Water from beverages (mL/day)	2380.0 (2203.0–2557.0)	2300.8 (2128.4–2473.1)	79.1	0.230	1462.2–1303.9
Water from food (mL/day)	459.0 (409.2–509.0)	437.7 (380.0–495.3)	21.3	0.398	551.5–508.9
Water intake (mL/day)	2839.0 (2649.1–3029.0)	2738.5 (2545.6–2931.3)	100.4	0.184	1685.8–1485.0
Water loss (mL/day)	3558.0 (3408.0–3708.0)	3606.1 (3455.2–3757.0)	−48.2	0.090	547.0–643.4
Water balance (mL/day)	−719.0 (−934.0–−504.0)	−855.4 (−1074.9–−636.0)	136.4	0.095	1848.1–1575.2

Results are presented as mean and confidence interval. *p* values derived through the Student’s *t* test.

## Supplementary Materials

The following are available online at https://www.mdpi.com/2072-6643/11/3/565/s1, Supplementary File S1: The hydration Status Questionnaire adolescent-young (HSQ-AY).
