# Supplementary material for: Adaptation and Validation of the Hydration Status Questionnaire in a Spanish Adolescent-Young Population: A Cross Sectional Study
Source: Nutrients. 2019 Mar 6;11(3):565. doi: 10.3390/nu11030565 (PMC6470467; doi:10.3390/nu11030565)
Supplement: Supplementary file 1 [file nutrients-11-00565-s001.pdf]

THE HYDRATION STATUS QUESTIONNAIRE

FOR ADOLESCENT-YOUNG POPULATION (HSQ-AY)

INSTRUCTION FOR COMPLETING THE QUESTIONNAIRE:

- Please read all the questions carefully and take your time while answering them.
- Concentrate while reading the questions and try to answer them as accurately as possible.
- Use an X to mark the answer that better represents your situation.
- Some questions can be answered with more than one answer, if so; this will be indicated in the column on the left.
- Some questions are accompanied by an asterisk (*). This means you will find an explanatory footnote on the bottom of the page.
- At the beginning of each section, you will find a description that will assist you in filling out the questions in that segment.

1. Personal Information

2. Medical history

Name				Surname			
Age		Course		Gender		Date	

**Do you have any disease or health problem?**	☐ Yes ☐ No
**Do you suffer from one of these illnesses?**<br>**MULTIPLE ANSWER POSSIBLE**	☐ Diabetes ☐ Urinary tract infection<br>☐ Bowel disease ☐ Heart failure<br>☐ Kidney disease ☐ Other: _________<br>(Please specify)
**Do you take any of these medications?**<br>**MULTIPLE ANSWER POSSIBLE**	☐ Laxative → Medication that accelerates intestinal transit. It is used to correct constipation.<br>☐ Diuretic → Medication to eliminate more fluids. They increase the urine production.<br>☐ Anti-inflammatory → Medication to reduce inflammation and pain, such as ibuprofen.<br>☐ Anti-hypertensive → Medication to control the tension.<br>☐ Other: ________<br>(Please specify)
**Do you take any nutritional supplement as for example vitamins or minerals?**	☐ Yes ☐ No<br>If the answer is affirmative, please indicate the type:
**Do you suffer from any of these symptoms?**<br>**MULTIPLE ANSWER POSSIBLE**	☐ Shivers ☐ Constipation ☐ Diarrhoea<br>☐ Dizziness ☐ Cramps ☐ Tachycardia<br>☐ Headache ☐ Asthenia/fatigue ☐ Dry mouth<br>☐ Lack of concentration
**Do you have difficulty performing any of the following actions?**<br>**MULTIPLE ANSWER POSSIBLE**	☐ Holding a cup ☐ Swallowing ☐ Opening a bottle

3. Hydration habits

**Do you usually have a bottle of water with you when you are outside?**	☐ Yes	☐ No
**Do you take a bottle of water when you go to school?**	☐ Yes	☐ No
**Do you drink water between meals?**	☐ Yes	☐ No
**Do you drink any water while performing physical activity?**	☐ Yes	☐ No
**Do you consume isotonic and/or energy drinks while performing physical activity?**	☐ Yes	☐ No
**Do you consume any fluid before feeling thirsty?**	☐ Yes	☐ No
**Do you like drinking water?**	☐ Yes	☐ No
**When you are thirsty, do you prefer to consume other beverages instead of water?**	☐ Yes<br>If the answer is affirmative, please indicate the type:________	☐ No
**Do you feel full after drinking fluids?**	☐ Yes	☐ No
**Do you know which is the daily water intake recommendation?**	☐ Yes<br>If the answer is affirmative, please indicate the amount:______	☐ No

4. Food and beverage frequency questionnaire

First column: Use an x to indicate the serving size that you usually consume
Second column: Use numbers to indicate the frequency of consumption; daily, per week or per month. (If the frequency is less than one per month do not answer the question)
Third column. Use an X to indicate the moments at which do you usually consume the product.

WATER. (It includes tap water, bottle water, fizzy water and water flavoured)

Indicate the habitual serving size	Indicate the number of glasses or bottles that you usually consume daily	Indicate the moments at which you usually consume water
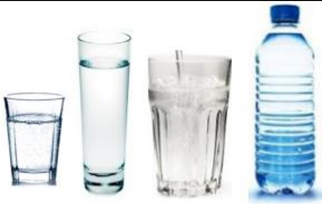<br>200 ml ☐ 250 ml ☐ 330 ml ☐ 500 ml ☐	1☐ 2☐ 3☐<br>4☐ 5☐ 6☐<br>7☐ 8☐ 9☐<br>10☐ 11☐ 12☐<br><br>If the amount is higher, please specify:	With breakfast ☐<br>With lunch ☐<br>With dinner ☐<br>Between hours ☐

JUICES (Naturals and bottled)

	Indicate the habitual serving size	Indicate the number of glasses or packages that you usually consume daily, per week or per month.				Indicate the moments of consumption
**Type 1: Fruits**	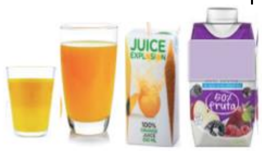<br>☐ ☐ ☐ ☐	Daily	Type 1	Type 2	Type 3	With breakfast ☐<br>With lunch ☐
**Type 2: Vegetables**		Per week	Type 1	Type 2	Type 3	With dinner ☐
**Type 3: With milk**		Per month	Type 1	Type 2	Type 3	Between hours ☐

SODAS

	Indicate the habitual serving size	Indicate the number of glasses or packages that you usually consume daily, per week or per month.				Indicate the moments of consumption
**Type 1: Nornal**	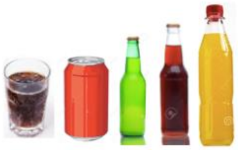<br>☐ ☐ ☐ ☐ ☐	Daily	Type 1	Type 2	Type 3	With breakfast ☐<br>With lunch ☐
**Type 2: Light**		Per week	Type 1	Type 2	Type 3	With dinner ☐
**Type 3: Zero**		Per month	Type 1	Type 2	Type 3	Between hours ☐

	Indicate the habitual serving size	Indicate the number of glasses or packages that you usually consume daily, per week or per month			Indicate the moments of consumption
Type 1: Isotonic beverages	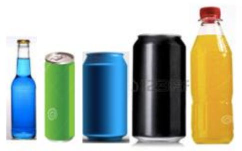 ☐ ☐ ☐ ☐ ☐	Daily	Type 1	Type 2	With breakfast ☐
Type 2: Energy beverages		Per week	Type 1	Type 2	With lunch ☐
					With dinner ☐
		Per month	Type 1	Type 2	
					Between hours ☐

MILK AND DAIRY PRODUCTS.

Milk → (It includes milk, milk with cacao, with honey and or with cereals. It not includes milk with coffee or milk in milkshakes).

	Indicate the habitual serving size	Indicate the number of glasses or packages that you usually consume daily, per week or per month				Indicate the moments of consumption
Type 1: Whole milk	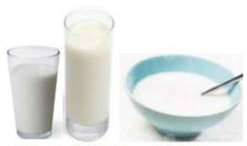 200 ml ☐ 250 ml ☐ 350 ml ☐	Daily	Type 1	Type 2	Type 3	With breakfast ☐
Type 2: Milk semi-skimmed		Per week	Type 1	Type 2	Type 3	With lunch ☐
Type 3: Milk skimmed		Per month	Type 1	Type 2	Type 3	With dinner ☐
						Between hours ☐

Milkshakes and yoghurts.

	Indicate the habitual serving size	Indicate the number of glasses or packages that you usually consume daily, per week or per month.				Indicate the moments of consumption
Type 1: Milkshakes	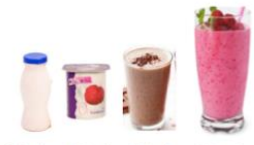 100 ml ☐ 125 ml ☐ 200 ml ☐ 330 ml ☐	Daily	Type 1	Type 2	Type 3	With breakfast ☐
						With lunch ☐
Type 2: Liquid yoghurt		Per week	Type 1	Type 2	Type 3	With dinner ☐
Type 3: yoghurt		Per month	Type 1	Type 2	Type 3	Between hours ☐

COFFEE

	Indicate the habitual serving size	Indicate the number of cups that you usually consume daily, per week or per month				Indicate the moments of consumption
**Type 1: Expresso coffee**	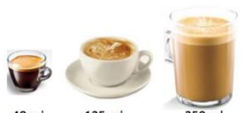 40 ml ☐ 125 ml ☐ 250 ml ☐	Daily	Type 1	Type 2	Type 3	With breakfast ☐
						With lunch ☐
**Type 2: Coffee with milk**		Per week	Type 1	Type 2	Type 3	With dinner ☐
**Type 3: American coffee (Large black coffee)**		Per month	Type 1	Type 2	Type 3	Between hours ☐

INFUSIONS

Indicate the habitual serving size	Indicate the number of cups that you usually consume daily, per week or per month		Indicate the moments of consumption
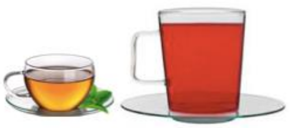 125 ml ☐ 250 ml ☐	Daily		With breakfast ☐
	Per week		With lunch ☐
	Per month		With dinner ☐
			Between hours ☐

ALCOHOLIC BEVERAGES

	Indicate the habitual serving size	Indicate the number of glasses that you usually consume daily, per week or per month				Indicate the moments of consumption
**Type 1: Sangría**	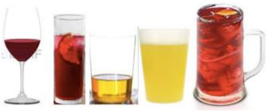 ☐ ☐ ☐ ☐ ☐	Daily	Type 1	Type 2	Type 3	With breakfast ☐
						With lunch ☐
**Type 2: Cider**		Per week	Type 1	Type 2	Type 3	With dinner ☐
**Type 3: Wine**		Per month	Type 1	Type 2	Type 3	Between hours ☐

Indicate the habitual serving size	Indicate the number of units that you usually consume daily, per week or per month.		Indicate the moments of consumption
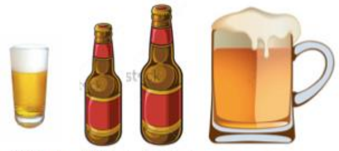 200 ml ☐ 250 ml ☐ 330ml ☐ 500 ml ☐	Daily		With breakfast ☐
	Per week		With lunch ☐
	Per month		With dinner ☐
			Between hours ☐

Indicate the habitual serving sizes	Indicate the number of units that you usually consume daily, per week or per month		Indicate the moments of consumption
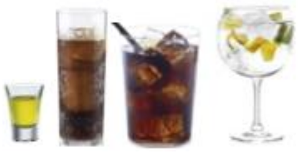 ☐ ☐ ☐ ☐	Daily		With breakfast ☐
	Per week		With lunch ☐
	Per month		With dinner ☐
			Between hours ☐

OTHER BEVERAGES

	Indicate the habitual serving sizes	Indicate the number of glasses that you usually consume daily, per week or per month.			Indicate the moments of consumption
**Type 1: Plant-based beverages (soy, oats, almonds)**	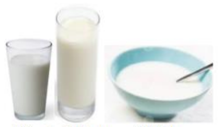 200 ml ☐ 250 ml ☐ 350 ml ☐	Daily	Type 1	Type 2	With breakfast ☐
					With lunch ☐
**Type 2: Horchata.**		Per week	Type 1	Type 2	With dinner ☐
		Per month	Type 1	Type 2	Between hours ☐

Do you usually consume some beverage that is not included in the questionnaire? If the answer is affirmative, please indicate it below.

TYPE	AMOUNT	FREQUENCY	MOMENTS

-
-
-
-
-

FRUITS. (It includes whole fruits. It not includes fruits juices or milkshakes)

Use an X to indicate the fruits that you usually consume.
In the column on the right, next to its corresponding letter, use a number to indicate how many times you consume this fruit (daily, per week or per month). Finally, indicate the habitual serving size
Example: If you usually consume strawberries ones a week, oranges one per month you should write: per week (A-1) per month (C-1).

A	B	C	D
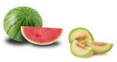 Watermelon/ melon	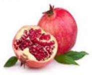 Pomegranate	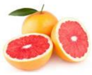 Grapefruit	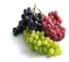 Grapes

Frequency of consumption	Daily	Per week	Per month
	A:<br>B:<br>C:<br>D:	A:<br>B:<br>C:<br>D:	A:<br>B:<br>C:<br>D:
Serving size	Big	Normal	Little

A	B	C	D
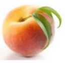 Peach/ Nectarine	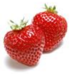 Strawberries	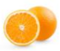 Orange	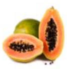 Papaya

Frequency of consumption	Daily	Per week	Per month
	A:<br>B:<br>C:<br>D:	A:<br>B:<br>C:<br>D:	A:<br>B:<br>C:<br>D:
Serving size	Big	Normal	Little

A	B	C	D
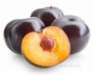 Apricot/ Plum	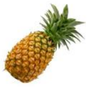 Pineapple	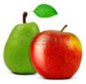 Peae/ apple	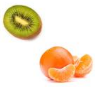 Kiwi/ tangerine

Frequency of consumption	Daily	Per week	Per month
	A:<br>B:<br>C:<br>D:	A:<br>B:<br>C:<br>D:	A:<br>B:<br>C:<br>D:
Serving size	Big	Normal	Little

A	B	C	D
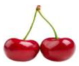 Cherries	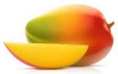 Mango	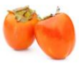 Khaki	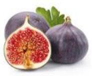 Figs

Frequency of consumption	Daily	Per week	Per month
	A:<br>B:<br>C:<br>D:	A:<br>B:<br>C:<br>D:	A:<br>B:<br>C:<br>D:
Serving size	Big	Normal	Little

VEGETABLES

<u>Use an X</u> to indicate the vegetables that you usually consume.
In the column on the right, next to it corresponding letter, use a number to indicate how many times you consume this fruit (daily, per week or per month). At last, indicate the habitual serving size
It not include vegetables soups or purees.

Example of serving size:

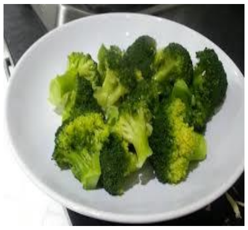

Main dish: Broccoli

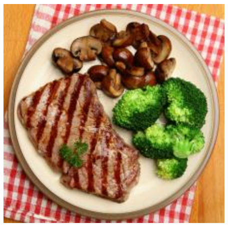

Garnish: Broccoli

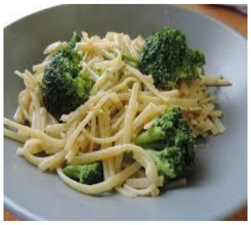

Ingredient of a dish: Pasta with cheese and broccoli

A	B	C	D
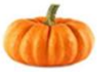 Zucchini/ Pumpkin	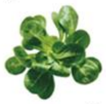 Lettuce/ Canon	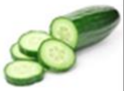 Cucumber	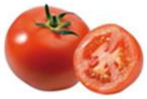 Tomato

Frequency of consumption	Daily	Per week	Per month
	A:<br>B:<br>C:<br>D:	A:<br>B:<br>C:<br>D:	A:<br>B:<br>C:<br>D:
Serving size	Dish	Garnish	Ingredient

A	B	C	D
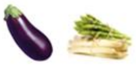 Eggplant/ Asparagus	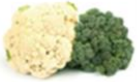 Cauliflower/ Broccoli	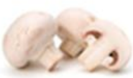 Mushroom	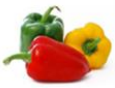 Pepper

Frequency of consumption	Daily	Per week	Per month
	A:<br>B:<br>C:<br>D:	A:<br>B:<br>C:<br>D:	A:<br>B:<br>C:<br>D:
Serving size	Dish	Garnish	Ingredient

A	B	C	D
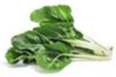 Chard/ Spinach	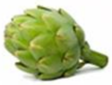 Artichoke/ Green bean	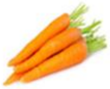 Carrot	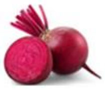 Beet

Frequency of consumption	Daily	Per week	Per month
	A:<br>B:<br>C:<br>D:	A:<br>B:<br>C:<br>D:	A:<br>B:<br>C:<br>D:
Serving size	Dish	Garnish	Ingredient

OTHER DISHES

	Amount	Frequency			Moments
		Daily	Per week	Per month	
**Gazpacho/ cold soups**	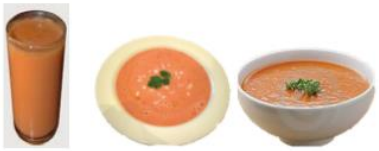<br>250 ml ☐ 350 ml ☐ 450 ml ☐				With breakfast ☐<br>With lunch ☐<br>With dinner ☐<br>Between hours ☐
**Soups**	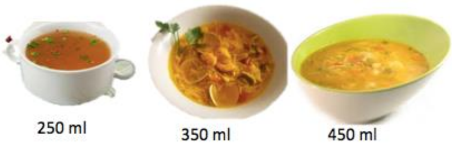<br>250 ml ☐ 350 ml ☐ 450 ml ☐				With breakfast ☐<br>With lunch ☐<br>With dinner ☐<br>Between hours ☐
**Vegetables purees**	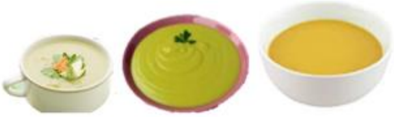<br>250 ml ☐ 350 ml ☐ 450 ml ☐				With breakfast ☐<br>With lunch ☐<br>With dinner ☐<br>Between hours ☐

5. Fluid losses

**How often do you urinate?**	☐ 1 time/day ☐ 8-10 times/day<br>☐ 2-4 times/day ☐ > 10 times/day<br>☐ 5-7 times/day
**How often do you defecate?**	☐ ≥1 time/day ☐ 1-2 times/week<br>☐ 5-6 times/week ☐ <1 time in 10 days<br>☐ 3-4 times/week
**Have you suffered from diarrhoea during the course of this study?**	☐ yes ☐ no
**How would you rate the quantity of sweat you produce on a regular day?**<br><br>**(Select one of these options to represent the quantity, with 1 being the minimum and 10 the maximum.)**	☐ 1 ☐ 2 ☐ 3 ☐ 4 ☐ 5<br>☐ 6 ☐ 7 ☐ 8 ☐ 9 ☐ 10
**How would you rate the quantity of sweat you produce while performing physical activity?**<br><br>**(Select one of these options to represent the quantity with 1 being the minimum and 10 the maximum.)**	☐ 1 ☐ 2 ☐ 3 ☐ 4 ☐ 5<br>☐ 6 ☐ 7 ☐ 8 ☐ 9 ☐ 10
